# Trajectory of Early Life Adiposity Among South Asian Children

**DOI:** 10.1001/jamanetworkopen.2025.4439

**Published:** 2025-04-10

**Authors:** Sandi M. Azab, Saba Naqvi, Talha Rafiq, Joseph Beyene, Wei Deng, Amel Lamri, Katherine M. Morrison, Koon Teo, Gillian Santorelli, John Wright, Natalie C. Williams, Russell J. de Souza, Gita Wahi, Sonia S. Anand

**Affiliations:** 1 Department of Medicine, McMaster University, Hamilton, Ontario, Canada; 2 Department of Pharmacognosy, Alexandria University, Alexandria, Egypt; 3 Department of Health Research Methods, Evidence, and Impact, McMaster University, Hamilton, Ontario, Canada; 4 Population Health Research Institute, Hamilton, Ontario, Canada; 5 Peter Boris Centre for Addictions Research, St Joseph’s Healthcare Hamilton, Hamilton, Ontario, Canada; 6 Department of Psychiatry and Behavioural Neurosciences, McMaster University, Ontario, Canada; 7 Department of Pediatrics, McMaster University, Hamilton, Ontario, Canada; 8 Bradford Institute for Health Research, Bradford Teaching Hospitals NHS Foundation Trust, Bradford, United Kingdom

## Abstract

**Importance:**

Measures of childhood adiposity merit investigation, particularly in individuals of South Asian descent.

**Objective:**

To investigate prenatal and childhood factors associated with the trajectory of adiposity in South Asian children, and the cumulative contribution of modifiable factors, such as diet and physical activity, on this trajectory.

**Design, Setting, and Participants:**

This cohort study was a prospective analysis of the South Asian Birth Cohort (START; 2011-2015) for discovery; and the Family Atherosclerosis Monitoring In Early Life (FAMILY; 2002-2009) in Ontario, Canada, and the Born in Bradford (BiB; 2008-2009) cohort in Bradford, UK, for validation. Mother-child pairs included 903 South Asian individuals (START), 675 White European individuals (FAMILY), and 1593 individuals (BiB), of which 52% were South Asian. Analysis was conducted from March 2020 to September 2024.

**Exposure:**

Maternal, infancy, and early childhood exposures.

**Main Outcomes and Measures:**

Adiposity, assessed by the sum of subscapular and triceps skinfold thicknesses (SSF) from birth to 3 years, aggregated to a single measure as total area under the growth curve (AUC for SSF); multivariable linear regression models to identify determinants of AUC for SSF; and a cumulative score to assess joint contribution of modifiable risk factors to AUC for SSF.

**Results:**

START included 903 children (456 female [50.5%]; mean [SD] maternal age, 30.2 [4.0] years; maternal mean [SD] prepregnancy body mass index [BMI], 23.8 [4.50]). Maternal sum of skinfold thicknesses (β = 0.80 [95% CI, 0.30-1.30] per 10 mm), gestational weight gain (β = 0.38 [95% CI, 0.02-0.74] per 5 kg), a health-conscious diet score (β = −0.68 [95% CI, −1.26 to −0.10] per 1 SD), and infant breastfeeding for the first year (β = −1.68 [95% CI, −2.94 to −0.42), as well as physical activity (β = −0.33 [95% CI, −0.57 to −0.09] per 30-min/d) and screen time (β = 0.49 [95% CI, 0.18-0.81] per 30-min/d) were each independently associated with AUC for SSF. These 6 early-life modifiable factors combined into a single score had a direct, graded association between number of factors and AUC for SSF (*P *for trend < .001). In the validation cohorts, maternal BMI, breastfeeding, and child physical activity were replicated and showed a similar graded association with AUC for SSF (*P *for trend < .001) when combined.

**Conclusions and Relevance:**

In this cohort study of South Asian children, 6 modifiable factors were associated with lower adiposity and combined into a single score. This score may be useful in clinical and public health settings to help mitigate childhood obesity in South Asian individuals and beyond.

## Key Points

**Question:**

What maternal and early life factors are associated with the trajectory of adiposity in South Asian children from birth to 3 years of age?

**Findings:**

In this cohort study of 903 children, longer breastfeeding, increased physical activity, and reduced screen time, in addition to optimizing maternal diet, adiposity, and weight gain during pregnancy, were associated with lasting favorable adiposity outcomes for South Asian children. There was a significant, graded and direct association between the number of early life modifiable exposures and childhood adiposity validated across 3 cohorts in 3171 South Asian and White European children.

**Meaning:**

These results suggest that selected modifiable factors can be combined into a single score, which may be useful in clinical and public health settings for risk assessment and targeted interventions to help curb childhood obesity.

## Teaser

This cohort study examines prenatal and childhood factors associated with the trajectory of adiposity in South Asian children from birth to age 3 years.

## Introduction

Childhood obesity is a progressing global health concern.^1,2,3,4,5,6,7,8^ In 2020, an estimated 40 million children worldwide under the age of 5 years were classified as either overweight or obese.^9,10^ There is an urgent call to implement evidence-based recommendations to treat childhood obesity,^11^ along a paucity of studies and mixed evidence of preschool or home-based interventions.^12^

Increases in childhood obesity are greatest in Asia, the Middle East, and Latin America.^13,14^ South Asian individuals, people who originate from the Indian subcontinent, are among the fastest-growing ethnic groups in North America. South Asian individuals’ higher prevalence of central adiposity, higher prevalence of cardiometabolic risk factors, and higher predisposition to subsequent diabetes and cardiovascular disease in adulthood merit further research to improve health equity.^15,16,17,18,19^ South Asian newborns and children are characterized by low birth weight, but proportionally higher body fat or central obesity (a so-called thin-fat phenotype) relative to White Europeans, and this pattern persists in 4th generation migrated populations.^20,21^ Higher visceral fat, when conserved, is associated with long-term metabolic, cardiovascular, and cognitive outcomes.^19,22,23^ Despite its wide use, body mass index (BMI; calculated as weight in kilograms divided by height in meters squared) can be a suboptimal measure of fat distribution, especially in South Asian individuals and more valid measures of adiposity (eg, skinfold thicknesses), should be investigated.^24,25,26,27,28^

An extensive body of literature points to the association of prenatal and perinatal factors with childhood obesity, including maternal nutrition and smoking status, maternal prepregnancy BMI and gestational weight gain, gestational diabetes, and child sleep and feeding patterns.^29,30,31,32,33,34,35^ The majority of studies have been conducted in White European mother-infant dyads, where ethnic inequalities in the literature of obesity warrant further research.^13,14^ Additionally, most studies have focused on a single time point to assess obesity (eg, at birth or at 1 year) rather than over the course of the child’s early years, where the latter approach may better capture a child’s dynamic and continuous growth^36^ and better predict future obesity risk than a single measurement.^37^ We hypothesized that early life modifiable factors (eg, breastfeeding or physical activity) may be associated with early life adiposity trajectory, which may help guide interventions and public health policies to mitigate childhood obesity. In the present investigation, we aimed to (1) determine the perinatal and early life factors associated with the trajectory of adiposity over the first 3 years of life among South Asian children and (2) examine the cumulative contribution of modifiable factors on this trajectory, with validation in external cohorts.

## Methods

### Study Design and Participants

To meet the call for health equity and to investigate adiposity in South Asian children, this study was set within an exclusively South Asian birth cohort (START). START was the discovery cohort used for the primary investigation and analyses, followed by validation of final findings in 2 external birth cohorts that included both South Asian and White European children for potentially wider generalizability and implication for practice and policy (eFigure 1 in Supplement 1). START is a prospective birth cohort study in the Peel Region of Ontario, Canada, which recruited 1012 South Asian mothers during the second trimester of a singleton pregnancy, and their newborns between 2011 and 2015; recruitment, inclusion and exclusion criteria were previously described.^14,38,39^ Of 1002 infants enrolled in START, 903 completed at least 1 follow-up visit at 1, 2, or 3 years, and were included in this analysis. Validation cohorts included (1) the Family Atherosclerosis Monitoring In Early Life (FAMILY) prospective birth cohort study,^40^ which recruited 857 predominantly White European families from Hamilton, Ontario, Canada, between 2002 and 2009, of which 675 children completed at least 1 follow-up visit at 1, 2, or 3 years and were included in this analysis, and (2) the Born in Bradford (BiB)^41^ prospective family cohort subsample study (BiB1000),^42^ which investigated the predictors and influences of health-related behaviors that lead to the development of childhood obesity. It recruited 1735 pregnancies from the city of Bradford, UK, between August 2008 and March 2009, of which 1593 singleton pregnancies completed at least 1 follow-up visit at 0.5, 1, 1.5, 2, or 3 years and were included in this analysis.^43^ Ethics approvals from McMaster Hamilton Integrated Research Ethics Board (START and FAMILY) and Bradford Research Ethics Committee (BiB) were obtained, as well as written informed consent from the legal guardians of all study participants. Ethnicity was self-reported on the appropriate study questionnaire. We followed the Strengthening the Reporting of Observational Studies in Epidemiology (STROBE) reporting guideline for cohort studies.

### Growth Trajectories Across First 3 Years of Life

Standard protocols were used to obtain child anthropometric measurements. Skinfold thickness, a surrogate measure of adiposity, was calculated as the sum of triceps and subscapular skinfold thicknesses (SSF) measured for each child at birth and at each follow-up visit. Measurements were taken in triplicate using skinfold calipers and recorded to the nearest 0.2 mm in START (Holtain Tanner/Whitehouse, UK) and to the nearest 0.5 mm in FAMILY (Lange, UK), whereas in BiB skinfold measures were taken on the left side of the body (Holtain Tanner/Whitehouse, UK). Length was measured using a regularly maintained and calibrated pediatric length board at birth, 1-year visit, and 2-year visit (length was not taken in BiB); and height at the 3-year visit was measured using a stadiometer. Infant birth weight was obtained from birth delivery reports, and weight at each follow-up visit was measured using an electronic scale. The primary outcome of our study was the overall trajectory of adiposity (ie, SSF from birth to 3 years of age). In the discovery cohort, the overall trajectory of BMI from birth to 3 years was derived as a comparator to SSF. To capture change over time, we calculated the cumulative area under the curve (AUC) for each child’s growth curve of SSF and BMI from birth to 3 years. We used trapezoid calculations to derive the mean of 2 anthropometric values at 2 successive visits, multiplied by the time difference between the 2 visits (eFigure 2 in Supplement 1). The purpose of using AUC was the use of a simplified approach as a crude continuous metric of a child’s growth trajectory summarizing serial measurements into a single value.^44^ A detailed description of the AUC derivation and AUC method justification can be found in the eMethods in Supplement 1.

### Maternal, Infancy, and Childhood Exposure Variables

We investigated established maternal factors and early life factors that were associated with the development of obesity and adiposity in the offspring. Infancy and childhood exposure variables were collected using a combination of measurements at delivery and questionnaires administered to the mothers during follow-up visits. For a complete list of exposure variables and their definitions, see the eMethods in Supplement 1.

### Statistical Analysis

Descriptive statistics are presented for each cohort as means and standard deviations for continuous variables, and as counts and percentages for categorical variables. To determine the factors associated with the trajectory of adiposity in South Asian children over the first 3 years of life, we first assessed the association of maternal, infancy, and childhood exposure variables (all variables listed in Table 1) with AUC for SSF (primary outcome) and AUC for BMI (comparator) in START using simple linear regression models. Variables showing association at α ≤ .10 were entered into a backward elimination selection procedure for each outcome separately to arrive at a parsimonious set of projections of AUC for SSF and AUC for BMI. We acknowledge potential limitations of backward elimination where collinear variables or mediators may be retained, and exclusion of variables based on statistical significance rather than causal importance may occur. Factors retained from the variable selection procedure were chosen for the final multivariable linear regression models and considered significant determinants of the outcome at *P* < .05 in complete case analyses (n = 722 for AUC for SSF and n = 760 for AUC for BMI). All final multivariable models were additionally adjusted for age of the child at the 3-year visit to further control for confounding from older children having a higher AUC of the growth curve. To model the incremental contribution of all identified modifiable factors on AUC for SSF in South Asian children, these factors were combined into a single score. To create this composite score, binary variables were created for 6 modifiable variables on AUC for SSF, where continuous variables were categorized using the cohort median as the cutoff and defining the variable as associated with lower risk if at or below the median. A secondary analysis included use of clinical recommendation guided cutoffs detailed in the eMethods in Supplement 1. The derived binary variables were summed into the overall score ranging from 0 to 6 (n = 805). A higher score was interpreted as lower risk for adiposity. For validation, we constructed this cumulative score in the independent cohorts including only those factors that were individually replicated (ie, statistically significant), with an overall score ranging from 0 to 3 in FAMILY (n = 661) and BiB (n = 1141). Using linear regression models, the association of the score with AUC for SSF was examined on a continuous and categorical scale for the score, adjusting for child sex and age at the 3-year visit. Subgroup analysis of the score based on sex was also conducted in START. All analyses were completed in R version 3.6.3 (R Foundation) from March 2020 to September 2024.

**Table 1.** Characteristics of Mothers and Children Studied in the START, FAMILY, and BiB Birth Cohorts

Characteristics during recruitment period	START (2011-2015)		FAMILY (2002-2009)		BiB1000 (2008-2009)	
	No. (%)	Total No.^a^	No. (%)	Total No.^a^	No. (%)	Total No.^a^
Maternal						
Age, mean (SD), y	30.2 (3.95)	903	32.8 (4.82)	675	27.1 (5.62)	1593
Ethnicity, South Asian	903 (100.0)	903	12 (2.0)	675	854 (52.2)	1593
Prepregnancy BMI, mean (SD)	23.8 (4.50)	901	26.5 (6.26)	663	25.8 (5.69)	1529
Gestational weight gain, mean (SD), kg	14.3 (7.91)	888	15.3 (5.76)	651	NA	NA
Skinfolds thickness, mean (SD), mm	49.8 (12.3)	895	55.2 (19.7)	661	26.51 (7.0)^b^	435
Gestational hypertension	24 (2.7)	903	23 (3.4)	675	125 (8.0)	1514
Gestational diabetes	335 (37.1)	899	96 (14.2)	633	133 (9.0)	1516
AUC for glucose, mean (SD), mmol × min	815 (173)	845	771 (147)	633	NR	NR
Social disadvantage^c^						
Low	349 (38.6)	777	505 (74.8)	658	600 (37.9)	1585
Moderate	304 (33.7)	777	117 (17.3)	658	716 (45.2)	1585
High	124 (13.7)	777	36 (5.3)	658	269 (16.9)	1585
Smoking history						
Never or no	896 (99.2)	902	102 (15.1)	235	1151 (72.0)^d^	1592
Quit before pregnancy	4 (0.4)	902	109 (16.1)	235	NA	1592
Quit during pregnancy	2 (0.2)	902	24 (3.6)	235	NA	1592
Currently smoking	0	902	0	235	210 (13.0)	1592
Plant-based diet, mean (SD)	1.39 (1.10)	903	0.206 (0.664)	675	NR	NR
Western diet, mean (SD)	−0.557 (0.613)	903	1.02 (1.18)	675	NR	NR
Health-conscious diet, mean (SD)^e^	−0.423 (0.795)	903	−0.757 (0.713)	675	NR	NR
Physical activity prior to pregnancy, mean (SD), min/d	21.5 (32.8)	897	39.8 (29.2)	512	NR	NR
Physical activity during pregnancy, mean (SD)	12.6 (21.2)	893	32.5 (22.9)	318	219 (13.8)^f^	1592
Infancy and childhood						
AUC for SSF, mean (SD), mm × y	53.84 (8.1)	903	53.14 (9.6)	675	53.58 (7.6)	1593
AUC for BMI, mean (SD), m^2^/kg × y	48.46 (4.75)	903	NA	NA	NA	NA
Gestational age, mean (SD), wk	39.1 (1.5)	902	38.9 (2.0)	675	NA	NA
Female	456 (50.5)	903	334 (49.5)	675	798 (50.5)	1579
Size for gestational age						
Average	706 (78.2)	897	536 (79.4)	674	1216 (77.9)	1561
Small	140 (15.5)	897	51 (7.6)	674	234 (15)	1561
Large	51 (5.6)	897	87 (12.9)	674	111 (7)	1561
Breastfeeding for the first year	510 (56.5)	822	209 (31.0)	658	208 (14.5)	1430
Age of solid food introduction, mean (SD), mo	5.23 (1.16)	754	4.84 (1.14)	673	4.61 (1.05)	1325
Age at 3 y visit, mean (SD), y	3.02 (0.139)	829	3.06 (0.101)	675	2.94 (0.24)	1216
Night sleep, mean (SD), h/night	9.85 (0.870)	864	10.9 (0.705)	673	11.89 (1.2)	1216
Physical activity, mean (SD), min/d^g^	196 (79.4)	851	245 (63.9)	673	211 (122)	1317
Screen time, mean (SD), min/d	110 (56.7)	840	132 (80)	674	82 (59.5)	1477
Ultra-processed foods consumption, mean (SD), % daily intake	16.7 (7.35)	759	28.5 (12.3)	667	NR	NR

Abbreviations: AUC, total area under the curve; BiB, Born in Bradford; FAMILY, Family Atherosclerosis Monitoring In Early Life; NA, not applicable; NR, not reported; START, South Asian Birth Cohort.

^a^ Total No. is the number of participants with complete data for numerical variables and the sum of all categories for categorical variables.

^b^ Triceps only.

^c^ Variable adapted to BiB definition which originally included 5 categories of socioeconomic position ranging from 1 (least deprived and most educated) to 5 (most deprived).

^d^ Smoking history in BiB was captured as whether mother ever regularly smoked, and whether mother smoked nowadays.

^e^ Health-conscious diet characterized by seafood, poultry, eggs, fruits, vegetables, and refined grains. (Refined grains include: cold cereals, pancakes, French toast, waffles, muffins, scones, croissants, puri, idli and dosa, parathas, breads, corn bread, soft pretzels, white rice, and noodles).

^f^ Maternal physical activity in BiB captured at baseline as general practice physical activity. Score categorized as inactive, moderately inactive, moderately active, active; data presented as No. (%) of active or moderately active categories combined.

^g^ Physical activity was measured differently and should not be compared between cohorts.

## Results

### Participants Characteristics

The START discovery cohort included 903 children born to mothers with a mean (SD) age of 30.2 (4.0) years and a mean (SD) prepregnancy BMI of 23.8 (4.5). The mean (SD) gestational age of START children was 39.1 (1.5) weeks, 140 (15.5%) were small for gestational age, 456 (50.5%) were female, and 510 (56.5%) were breastfed for the entire first year of life. Across the first 3 years of life, START children exercised for a mean of 196 min/d, slept for a mean of 9.85 hours/night, and were exposed to a mean of 110 min/d of screen time, comparable to screen time in US children aged 3 years and younger.^45^ Demographic and clinical parameters of the mother-child dyads in START, FAMILY, and BiB are summarized in Table 1.

### Exposure Factors Associated With Adiposity in South Asian Children (START Cohort)

**Figure 1.**
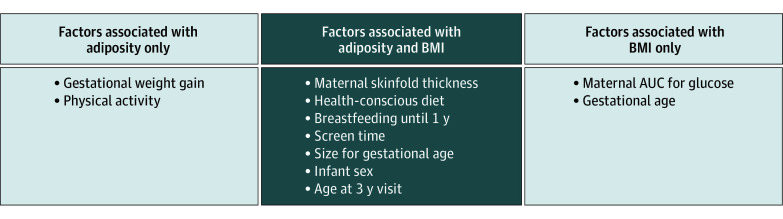
Diagram Depicting Factors Associated With Adiposity Compared With Body Mass Index (BMI) in South Asian Children in START AUC indicates total area under the curve; START, South Asian Birth Cohort.

Exposures univariately associated (α ≤ .10) with SSF_AUC_ in South Asian children, were maternal prepregnancy BMI, gestational weight gain, skinfold thickness, and a maternal health-conscious dietary pattern, and child size for gestational age, breastfeeding, age of solids introduction, physical activity, and screen time exposure (eTable 1 in Supplement 1). Of these, a health-conscious dietary pattern, small for gestational age, breastfeeding, and physical activity were inversely associated with AUC for SSF. In the final multivariable model, 6 modifiable and 2 nonmodifiable factors remained significantly associated with AUC for SSF in South Asian children (as highlighted in Table 2). Each 10 mm higher maternal skinfold thickness during pregnancy and each 5 kg higher maternal gestational weight gain were associated with 0.80 and 0.38-unit higher AUC for SSF in the offspring, respectively. Each 1-SD higher health-conscious diet factor score (characterized by seafood, poultry, eggs, fruits, vegetables, and refined grains) was associated with a 0.68 lower AUC for SSF. Children who were born small for gestational age had a 3.03-unit lower AUC for SSF compared with those born average for gestational age, and children who were breastfed through the first year of life had a 1.68-unit lower AUC for SSF compared with those who were breastfed for shorter time or not breastfed at all. Lastly, a 30-minute higher child physical activity was associated with a 0.33 lower AUC for SSF and a 30-minute higher screen time exposure was associated with a 0.49 higher AUC for SSF. Several exposures associated with AUC for SSF were also found to be associated with AUC for BMI, whereas other exposures were unique as shown in Figure 1 and eTable 2 in Supplement 1.

**Table 2.** Multivariable Model Estimates of Adiposity in South Asian Children (START)

Exposure	AUC for SSF (SE) [95% CI]^a^	*P* value
Age at the 3-y visit, y	18.44 (2.29) [13.94 to 22.94]	<.001
Small for gestational age	−3.03 (0.85) [−4.71 to −1.36]	<.001
Large for gestational age	1.98 (1.35) [−0.67 to 4.63]	.14
Maternal sum of skinfolds (10 mm)	0.80 (0.25) [0.30 to 1.30]	.002
Screen time (30 min/d)	0.49 (0.16) [0.18 to 0.81]	.002
Physical activity (30 min/d)	−0.33 (0.12) [−0.57 to −0.09]	.008
Breastfeeding at 1 y	−1.68 (0.64) [−2.94 to −0.42]	.009
Health-conscious diet (1 SD)	−0.68 (0.29) [−1.26 to −0.10]	.02
Infant sex (female)	1.41 (0.61) [0.22 to 2.60]	.02
Gestational weight gain (5 kg)	0.38 (0.18) [0.02 to 0.74]	.04

Abbreviations: AUC, total area under the growth curve; SSF, sum of triceps and subscapular skinfold thickness.

^a^ Multivariable linear regression model with AUC for SSF as outcome, adjusting for child sex and age at the 3-year visit based on complete case analysis (n = 722).

### Combined Contribution of Significant Modifiable Exposure Factors

**Figure 2.**
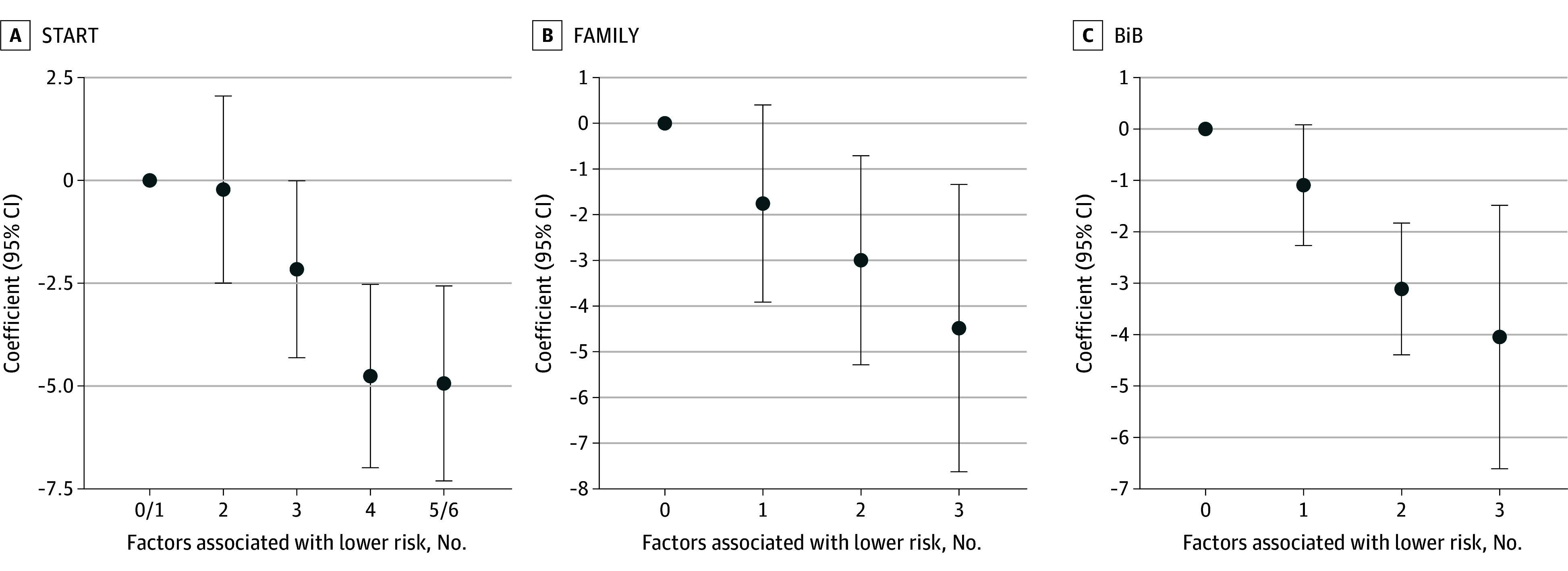
Combined Contribution on Adiposity in Children, by Study Coefficients and 95% CI for adiposity (total area under the growth curve [AUC] for sum of triceps and subscapular skinfold thickness [SSF]) in the South Asian Birth Cohort (START), Family Atherosclerosis Monitoring In Early Life (FAMILY), and Born in Bradford (BiB) children, adjusting for child sex and age at the 3-year visit, according to the number/score of modifiable factors associated with lower risk. Score was calculated as follows in START: 1 point for: less than or equal to median of maternal skinfold thickness, less than or equal to median of maternal gestational weight gain, greater than or equal to median of maternal health-conscious diet score, breastfeeding for 1 year, less than or equal to the median of physical activity, less than the median of screen time exposure. Score was calculated as follows in FAMILY and BiB: 1 point for less than or equal to 25 of maternal body mass index (calculated as weight in kilograms divided by height in meters squared), breastfeeding for 1 year, greater than or equal to median of physical activity.

The cumulative contributions of the significant modifiable factors associated with the trajectory of adiposity were combined into a single score from 0 to 6, reflecting lower risk. Only 9% of children (73 of 805) had 0 or 1 factor associated with lower risk, 21% (167 of 805) had 2 factors, 32% (258 of 805) had 3 factors, 23% (186 of 805) had 4 factors, and 15% (121 of 805) had 5 or all 6 factors associated with lower risk. There was an inverse, graded association between the number of modifiable factors and AUC for SSF outcome (*P *for trend < .001) as shown in Table 3, Figure 2; eTables 3 and 4 in Supplement 1 for sex-stratified analysis. On a continuous scale, for each additional factor associated with lower risk, child AUC for SSF on average decreased by 1.5 (95% CI, −1.96 to −1.04) units (*P* < .001). Results remained consistent using clinical recommendation guided cutoffs (eTable 5 and eFigure 3 in Supplement 1).

**Table 3.** Combined Contribution of Factors Associated With Lower Risk of Adiposity in South Asian Children in START

No. of early life factors associated with lower risk of adiposity	No.	AUC for SSF (95% CI)^a^	*P* value
0 or 1	73	0	NA
2	167	−0.22 (−2.50 to 2.06)	.85
3	258	−2.16 (−4.31 to −0.01)	.05
4	186	−4.78 (−7.01 to −2.55)	<.001
5 or 6	121	−4.93 (−7.31 to −2.56)	<.001
β-trend continuous^b^	805	−1.50 (−1.96 to −1.04)	<.001

Abbreviations: AUC, total area under the growth curve; NA, not applicable; SSF, sum of triceps and subscapular skinfold thickness; START, South Asian Birth Cohort.

^a^ Score was calculated as follows in START: 1 point for less than or equal to median of maternal skinfold thickness, less than or equal to median of maternal gestational weight gain, greater than or equal to median of maternal health-conscious diet score, breastfeeding for 1 year, greater than or equal to median of physical activity, less than or equal to median of screen time exposure. Multivariable linear regression model with AUC for SSF as outcome, adjusting for child sex and age at the 3-year visit based on complete case analysis (n = 805).

^b^ *P* value for trend determined by linear regression models of AUC for SSF on continuous factor score.

### Validation of the Combined Score in External Cohorts

We aimed to validate the single combined score of modifiable factors in the FAMILY (85% White European) and BiB (52% South Asian) independent birth cohorts. Out of the 6 modifiable factors in START, maternal adiposity as measured by BMI, breastfeeding, and childhood physical activity were replicated in both cohorts. A similar inverse graded association between the number of modifiable risk factors (score from 0 to 3) and AUC for SSF outcome (*P* for trend < .001) was observed as shown in Figure 2 and eTables 6 and 7 in Supplement 1.

## Discussion

We identified early life factors associated with the trajectory of adiposity in the first 3 years of life among South Asian children. We derived an AUC metric for adiposity, which captures the overall trajectory of adiposity from birth to 3 years of age, by 1 outcome: AUC for SSF. Maternal adiposity, gestational weight gain, and a health-conscious diet pattern, as well as child sex, size for gestational age, breastfeeding for the first year, physical activity, and screen time, were independently associated with AUC for SSF among South Asian children. We found that modifiable risk factors could be combined into a single score that could be used for risk assessment and validated this score in independent cohorts of White European and South Asian children.

A systematic review of 38 studies reported a consistent association between maternal prepregnancy BMI and offspring overweight.^46^ Maternal overweight or obesity before pregnancy^47^ and gestational weight gain have been previously shown to be associated with greater childhood adiposity, consistent with what we have observed in South Asian mother-child pairs.^48,49^ Moreover, a maternal health-conscious diet during and around pregnancy characterized by poultry, eggs, fruits, vegetables, seafood, and refined grains was associated with lower trajectory of adiposity in South Asian children. In a cohort of 764 mother-infant pairs (60% non-Hispanic White), Starling et al^30^ found that a maternal dietary pattern characterized by eggs, starchy vegetables, and nonwhole grains was associated with greater newborn adiposity, while a pattern characterized by poultry, nuts, and whole grains was not.^30^ Further research is warranted that considers ethnic differences and cooking methods for different cultures when studying the effect of maternal diet on child health.^16,29^ In this study, we did not find evidence of an association between gestational diabetes and offspring adiposity (AUC for SSF) despite a trend for an association between gestational diabetes and offspring body size (AUC for BMI) and evidence of association in other studies.^33,50^ Furthermore, maternal smoking has been associated with childhood obesity,^31,43,51^ but we could not test this association due to the low prevalence of smoking (<1%) in South Asian women in START.

In terms of infancy and childhood exposure factors, breastfeeding for 12 months or more—a cutoff used in Project Viva (a prospective, observational cohort study of gestational factors, and offspring health)^52^—was associated with a favorable adiposity trajectory in South Asian as well as White European children. The evidence for breastfeeding and lower risk for childhood overweight is mixed,^43^ however, recent systematic reviews suggest a breastfeeding is associated with a lower risk for childhood overweight.^50^ Weyermann et al^53^ found that breastfeeding for more than 6 months was associated with lower odds of overweight at 2 years of age; potentially suggesting a delayed benefit, similar to our results of breastfeeding in the first year being associated with favorable adiposity trajectory up to 3 years of age.

Daily physical activity among children in early life, at an age where mobility is still developing, was significantly associated with lower adiposity (and not BMI) in South Asian children as well as in White European children. A systematic review of early life determinants of obesity concluded that less than 30 minutes of daily physical activity could be a risk factor for later overweight and obesity.^51^ However, most studies pertained to an older age range (3 to 11 years of age),^54,55,56^ whereas our study highlighted an association of higher physical activity within the first 3 of life with lower adiposity trajectory. This finding remained consistent across 3 independent cohorts—measured in 3 different ways—and underscores the potential benefit of prioritizing early life physical activity.

Given the various developmental determinants of childhood adiposity, it is valuable to target multiple modifiable factors for effective prevention.^57,58,59^ Evaluating the combined contribution of the 6 aforementioned modifiable maternal and postnatal exposures on the trajectory of adiposity in South Asian children, a graded cumulative effect was found, where having any 4 factors associated with lower risk (or all 3 factors associated with lower risk in FAMILY and BiB) seemed to have maximal gains. Such messaging is urgently needed, where we currently face an alarming rise in obesity and type 2 diabetes in young populations with overrepresentation from South Asian ethnic groups.^60^

### Implication for Practice and Policy

Our findings can help guide clinical assessment and public health promotion to help mitigate adiposity, specifically in high-risk ethnicities, such as South Asian individuals.^61^ In this study, we presented a translation of findings into a multicomponent score for implementation in population health. This score may be useful in clinical and public health settings to identify children in early life at greatest risk and target interventions that can modify these risk factors. This can be operationalized in the form of a risk assessment tool for referring families to counselling or intensive behavioral interventions for those having less than 3 factors associated with lower risk. This work can also aid in the design of evidence-based research interventions such as the global Healthy Life Trajectories Initiative (HeLTI) targeted to improve the health of children from preconception through pregnancy and into early childhood.^62,63^ Next steps should be targeted toward dissemination of the generated knowledge to parents, and toward shaping actionable recommendations and policies to support healthy life trajectories (eg, subsidized maternal nutrition programs, promoting breastfeeding and access to breast pumping equipment, and early childhood physical activity interventions).^11^ However, we note that these interacting and multifaceted exposures are implicitly connected to wider, structural and social determinants of health, and require multiple system-wide interventions collectively to attain primary prevention.^64,65^ Moreover, maternal prepregnancy weight might be beyond the individual’s control and only be modifiable with the aid of pharmacological interventions.

### Strengths and Limitations

Our study has several strengths, which include its large sample size (3171 children) in 3 well-characterized longitudinal birth cohorts. By investigating adiposity in South Asian children focusing our primary analysis in START, we aimed to address ethnic disparities in childhood obesity.

This study has limitations that should also be considered. Child physical activity was measured by maternal report rather than accelerometry. Dietary assessment was also self-reported. These self-reported behaviors might have introduced bias due to recall error, social desirability bias, or differential reporting among families of children with or without obesity.^66,67,68^ Furthermore, we cannot rule out the possibility of unmeasured confounders such as paternal factors or genetic predispositions. Additionally, this is an observational study, and it does not allow distinction between cause and effect with the outcome of adiposity. Nevertheless, there was a temporal relation, whereby many of the factors we investigated preceded the outcome.

## Conclusions

This cohort study highlights the association of higher maternal diet quality, lower adiposity and gestational weight gain, and longer breastfeeding duration, as well as increased physical activity and reduced screen time in early life with lower adiposity in South Asian children. Our findings suggest that early interventions to change these risk factors could reduce childhood obesity.

## Notes

Supplement 1. **eTable 1.** Factors Univariately Associated With Adiposity (SSF_AUC_) in South Asian Children (START) **eTable 2.** Factors Associated With Obesity (BMI_AUC_) in South Asian children (START) **eTable 3.** Combined Contribution of Protective Factors of Adiposity (SSF_AUC_) in South Asian Boys in START **eTable 4.** Combined Contribution of Protective Factors of Adiposity (SSF_AUC_) in South Asian Girls in START **eTable 5.** Combined Contribution of Protective Factors of Adiposity (SSF_AUC_) in South Asian Children in START Based on Clinical Recommendation Guided Cutoffs **eTable 6.** Combined Contribution of Protective Factors of Adiposity (SSF_AUC_) in FAMILY **eTable 7.** Combined Contribution of Protective Factors of Adiposity (SSF_AUC_) in BiB **eFigure 1.** Consort Chart for Study Design in START, FAMILY, and BiB1000 **eFigure 2.** A. Illustration of Trapezoids for SSF_AUC_ Derivation. B. Distribution of the SSF_AUC_ Metric in START (Blue), FAMILY (Grey), and BiB (Red) Children **eFigure 3.** Combined Contribution on Adiposity in Children in START, Based on Clinical Recommendation Guided Cut-Offs
eMethods.

Supplement 2.
Data Sharing Statement
