# Supplementary material for: Trajectory of Early Life Adiposity Among South Asian Children
Source: JAMA Netw Open. 2025 Apr 10;8(4):e254439. doi: 10.1001/jamanetworkopen.2025.4439 (PMC11986779; doi:10.1001/jamanetworkopen.2025.4439)
Supplement: Supplement 1. — eTable 1. Factors Univariately Associated With Adiposity (SSFAUC) in South Asian Children (START) eTable 2. Factors Associated With Obesity (BMIAUC) in South Asian children (START) eTable 3. Combined Contribution of Protective Factors of Adiposity (SSFAUC) in South Asian Boys in START eTable 4. Combined Contribution of Protective Factors of Adiposity (SSFAUC) in South Asian Girls in START eTable 5. Combined Contribution of Protective Factors of Adiposity (SSFAUC) in South Asian Children in START Based on Clinical Recommendation Guided Cutoffs eTable 6. Combined Contribution of Protective Factors of Adiposity (SSFAUC) in FAMILY eTable 7. Combined Contribution of Protective Factors of Adiposity (SSFAUC) in BiB eFigure 1. Consort Chart for Study Design in START, FAMILY, and BiB1000 eFigure 2. A. Illustration of Trapezoids for SSFAUC Derivation. B. Distribution of the SSFAUC Metric in START (Blue), FAMILY (Grey), and BiB (Red) Children eFigure 3. Combined Contribution on Adiposity in Children in START, Based on Clinical Recommendation Guided Cut-Offs eMethods. [file jamanetwopen-e254439-s001.pdf]

# Supplemental Online Content

Azab SM, Naqvi S, Rafiq T, et al. Trajectory of early life adiposity among South Asian children. *JAMA Netw Open.* 2025;8(4):e254439.
doi:10.1001/jamanetworkopen.2025.4439

**eTable 1.** Factors Univariately Associated With Adiposity ($SSF_{AUC}$) in South Asian Children (START)

**eTable 2.** Factors Associated With Obesity ($BMI_{AUC}$) in South Asian children (START)

**eTable 3.** Combined Contribution of Protective Factors of Adiposity ($SSF_{AUC}$) in South Asian Boys in START

**eTable 4.** Combined Contribution of Protective Factors of Adiposity ($SSF_{AUC}$) in South Asian Girls in START

**eTable 5.** Combined Contribution of Protective Factors of Adiposity ($SSF_{AUC}$) in South Asian Children in START Based on Clinical Recommendation Guided Cutoffs

**eTable 6.** Combined Contribution of Protective Factors of Adiposity ($SSF_{AUC}$) in FAMILY

**eTable 7.** Combined Contribution of Protective Factors of Adiposity ($SSF_{AUC}$) in BiB

**eFigure 1.** Consort Chart for Study Design in START, FAMILY, and BiB1000

**eFigure 2.** A. Illustration of Trapezoids for $SSF_{AUC}$ Derivation. B. Distribution of the $SSF_{AUC}$ Metric in START (Blue), FAMILY (Grey), and BiB (Red) Children

**eFigure 3.** Combined Contribution on Adiposity in Children in START, Based on Clinical Recommendation Guided Cut-Offs

**eMethods.**

This supplemental material has been provided by the authors to give readers additional information about their work.

© 2025 Azab SM et al. *JAMA Network Open*.

eTable 1. Factors univariately associated with adiposity ($SSF_{AUC}$) in South Asian children (START)

| Exposure | Estimate | Std. Error | 95% CI | p- |
|---|---|---|---|---|
| Maternal prepregnancy BMI | 0.11 | 0.06 | (-0.01 to 0.23) | 0.07 |
| Maternal sum of skinfolds | 0.08 | 0.02 | (0.04 to 0.13) | <0.001 |
| Gestational weight gain | 0.08 | 0.03 | (0.01 to 0.15) | 0.02 |
| Health-Conscious diet | -0.83 | 0.34 | (-1.49 to -0.17) | 0.01 |
| Small for gestational age | -2.81 | 0.74 | (-4.27 to -1.35) | <0.001 |
| Large for gestational age | 2.44 | 1.16 | (0.16 to 4.73) | 0.04 |
| Breastfeeding at 1yr | -2.01 | 0.59 | (-3.17 to -0.85) | 0.001 |
| Age of solids introduction (m) | 0.72 | 0.27 | (0.20 to 1.24) | 0.007 |
| Physical activity (h/d) | -0.01 | 0.00 | (-0.01 to –0.00) | 0.04 |
| Screen time (min/d) | 0.89 | 0.30 | (0.31 to 1.48) | 0.003 |

95% CI: confidence intervals, p-: p-value for statistical significance. Univariable linear regression model with $SSF_{AUC}$ as outcome.

© 2025 Azab SM et al. *JAMA Network Open*.

eTable 2. Factors associated with obesity ($BMI_{AUC}$) in South Asian children (START)

| Exposure | Estimate | Std. Error | 95% CI | p- |
|---|---|---|---|---|
| Maternal sum of skinfolds (10 mm) | 0.47 | 0.13 | (0.20 to 0.72) | <0.001 |
| Maternal AUC glucose (1 SD) | 0.34 | 0.16 | (0.03 to 0.66) | 0.03 |
| Health-Conscious diet (1 SD) | -0.41 | 0.15 | (-0.71 to -0.11) | 0.008 |
| Gestational age (1 week) | 0.32 | 0.11 | (0.11 to 0.53) | 0.002 |
| Small for gestational age | -2.54 | 0.44 | (-3.40 to -1.68) | <0.001 |
| Large for gestational age | 1.95 | 0.66 | (0.65 to 3.25) | 0.003 |
| Breastfeeding at 1yr | -0.62 | 0.32 | (-1.25 to 0.00) | 0.05 |
| Screen time (30 min/d) | 0.28 | 0.08 | (0.12 to 0.44) | 0.001 |
| Infant sex (female) | -0.91 | 0.31 | (-1.51 to -0.31) | 0.003 |
| Age at the 3yr visit (y) | 15.65 | 1.14 | (13.41 to 17.89) | <0.001 |

95% CI: confidence intervals, p-: p-value for statistical significance. Multivariable linear regression model with $SSF_{AUC}$ as outcome, adjusting for child sex and age at the 3 yr visit based on complete case analysis n=760.

© 2025 Azab SM et al. *JAMA Network Open*.

eTable 3. Combined contribution of protective factors of adiposity ($SSF_{AUC}$) in South Asian boys in START

| # of early life protective factors | N | Estimate | 95% CI | p- |
|---|---|---|---|---|
| 0 or 1 | 41 | 0 | - | - |
| 2 | 85 | -0.70 | (-3.67 to 2.27) | 0.64 |
| 3 | 115 | -3.06 | (-5.88 to -0.23) | 0.03 |
| 4 | 97 | -3.46 | (-6.35 to -0.56) | 0.02 |
| 5 or 6 | 57 | -5.43 | (-8.59 to -2.28) | <0.001 |
| beta-trend* continuous | 395 | -1.30 | (-1.91 to -0.68) | <0.001 |

Score was calculated as follows in START: one point for: ≤ median of maternal skinfold thickness, ≤ median of maternal gestational weight gain, ≥ median of maternal health-conscious diet score, breastfeeding for 1 yr, ≥ median of physical activity, ≤ median of screen time exposure.
95% CI: confidence intervals, p-: p-value for statistical significance. Multivariable linear regression model with $SSF_{AUC}$ as outcome, adjusting for child sex and age at the 3 yr visit based on complete case analysis n=395.
* P-trend determined by linear regression models of $SSF_{AUC}$ on continuous factor score.

© 2025 Azab SM et al. *JAMA Network Open*.

eTable 4. Combined contribution of protective factors of adiposity ($SSF_{AUC}$) in South Asian girls in START

| # of early life protective factors | N | Estimate | 95% CI | p- |
|---|---|---|---|---|
| 0 or 1 | 32 | 0 | - | - |
| 2 | 82 | 0.35 | (-3.19 to 3.88) | 0.85 |
| 3 | 143 | -1.38 | (-4.7 to 1.95) | 0.42 |
| 4 | 90 | -6.07 | (-9.54 to -2.59) | <0.001 |
| 5 or 6 | 64 | -4.44 | (-8.06 to -0.82) | 0.02 |
| beta-trend* continuous | 411 | -1.73 | (-2.41 to -1.05) | <0.001 |

Score was calculated as follows in START: one point for: ≤ median of maternal skinfold thickness, ≤ median of maternal gestational weight gain, ≥ median of maternal health-conscious diet score, breastfeeding for 1 yr, ≥ median of physical activity, ≤ median of screen time exposure.
95% CI: confidence intervals, p-: p-value for statistical significance. Multivariable linear regression model with $SSF_{AUC}$ as outcome, adjusting for child sex and age at the 3 yr visit based on complete case analysis n=411.
* P-trend determined by linear regression models of $SSF_{AUC}$ on continuous factor score.

© 2025 Azab SM et al. *JAMA Network Open*.

eTable 5. Combined contribution of protective factors of adiposity ($SSF_{AUC}$) in South Asian children in START based on clinical recommendation guided cutoffs

| **# of early life protective factors** | N | Estimate | 95% CI | p- |
|---|---|---|---|---|
| 0 or 1 | 108 | 0 | - | - |
| 2 | 228 | -0.78 | (-2.70 to -1.14) | 0.43 |
| 3 | 266 | -1.91 | (-3.78 to -0.04) | 0.046 |
| 4 | 151 | -2.47 | (-4.52 to -0.42) | 0.018 |
| 5 or 6 | 50 | -3.80 | (-6.53 to -1.06) | 0.007 |
| beta-trend* continuous | 803 | -0.90 | (-1.40 to -0.41) | <0.001 |

95% CI: confidence intervals, p-: p-value for statistical significance. Multivariable linear regression model with $SSF_{AUC}$ as outcome, adjusting for child sex and age at the 3 yr visit based on complete case analysis n=803. * P-trend determined by linear regression models of $SSF_{AUC}$ on continuous factor score.

© 2025 Azab SM et al. *JAMA Network Open*.

eTable 6. Combined contribution of protective factors of adiposity ($SSF_{AUC}$) in FAMILY

| # of early life protective factors | N | Estimate | 95% CI | p- |
|---|---|---|---|---|
| 0 | 98 | 0 | - | - |
| 1 | 303 | -1.76 | (-3.92 to 0.40) | 0.11 |
| 2 | 207 | -3.0 | (-5.29 to -0.71) | 0.01 |
| 3 | 53 | -4.49 | (-7.63 to -1.34) | 0.005 |
| beta-trend* continuous | 661 | -1.44 | (-2.32 to -0.56) | 0.001 |

95% CI: confidence intervals, p-: p-value for statistical significance. Multivariable linear regression model with $SSF_{AUC}$ as outcome, adjusting for child sex and age at the 3 yr visit based on complete case analysis n=661.
* P-trend determined by linear regression models of $SSF_{AUC}$ on continuous factor score.

© 2025 Azab SM et al. *JAMA Network Open*.

eTable 7. Combined contribution of protective factors of adiposity ($SSF_{AUC}$) in BiB

| # of early life protective factors | N | Estimate | 95% CI | p- |
|---|---|---|---|---|
| 0 | 233 | 0 | - | - |
| 1 | 540 | -1.09 | (-2.27 to 0.08) | 0.07 |
| 2 | 328 | -3.11 | (-4.39 to -1.83) | <0.0001 |
| 3 | 40 | -4.05 | (-6.61 to -1.49) | 0.002 |
| beta-trend* continuous | 1141 | -1.54 | (-2.11 to -0.98) | <0.001 |

95% CI: confidence intervals, p-: p-value for statistical significance. Multivariable linear regression model with $SSF_{AUC}$ as outcome, adjusting for child sex and age at the 3 yr visit based on complete case analysis n=1141.
* P-trend determined by linear regression models of $SSF_{AUC}$ on continuous factor score.

© 2025 Azab SM et al. *JAMA Network Open*.

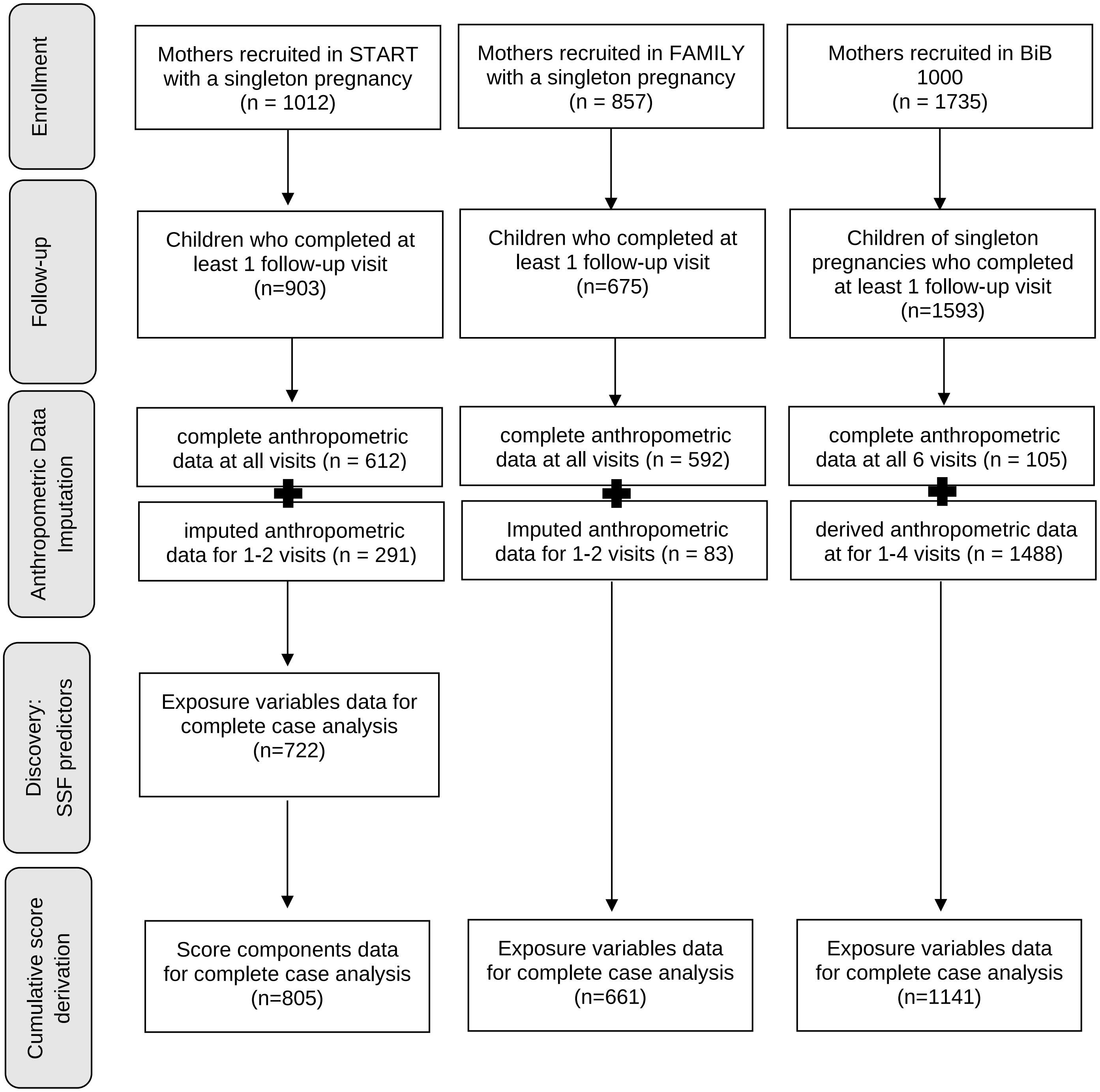


**eFigure 1: Consort chart for study design in START, FAMILY, and BiB1000**

© 2025 Azab SM et al. *JAMA Network Open*.

A

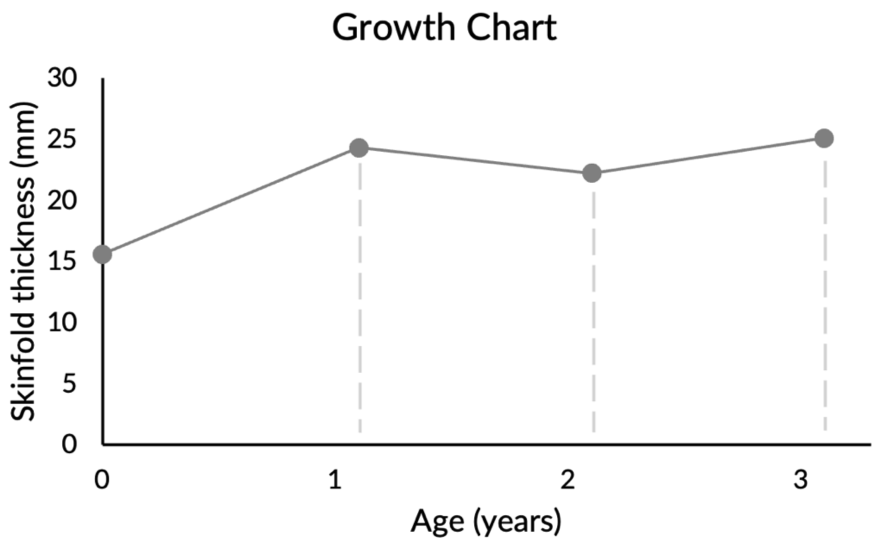


B

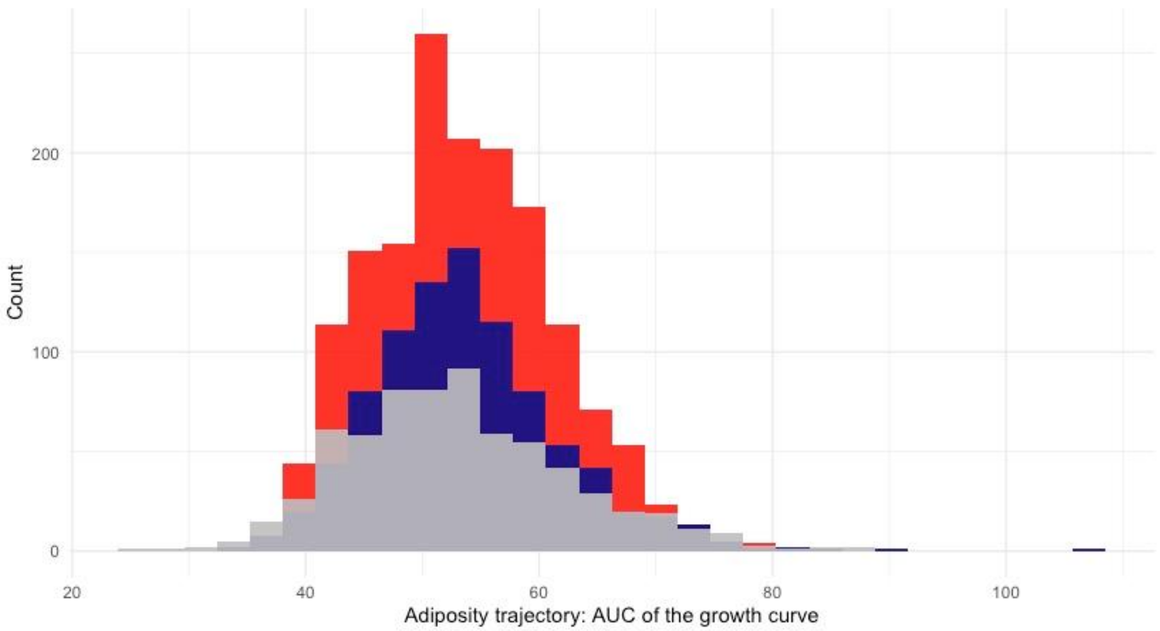


**eFigure 2. A. Illustration of trapezoids for $SSF_{AUC}$ derivation. B. Distribution of the $SSF_{AUC}$ metric in START (blue), FAMILY (grey), and BiB (red) children**

© 2025 Azab SM et al. *JAMA Network Open*.

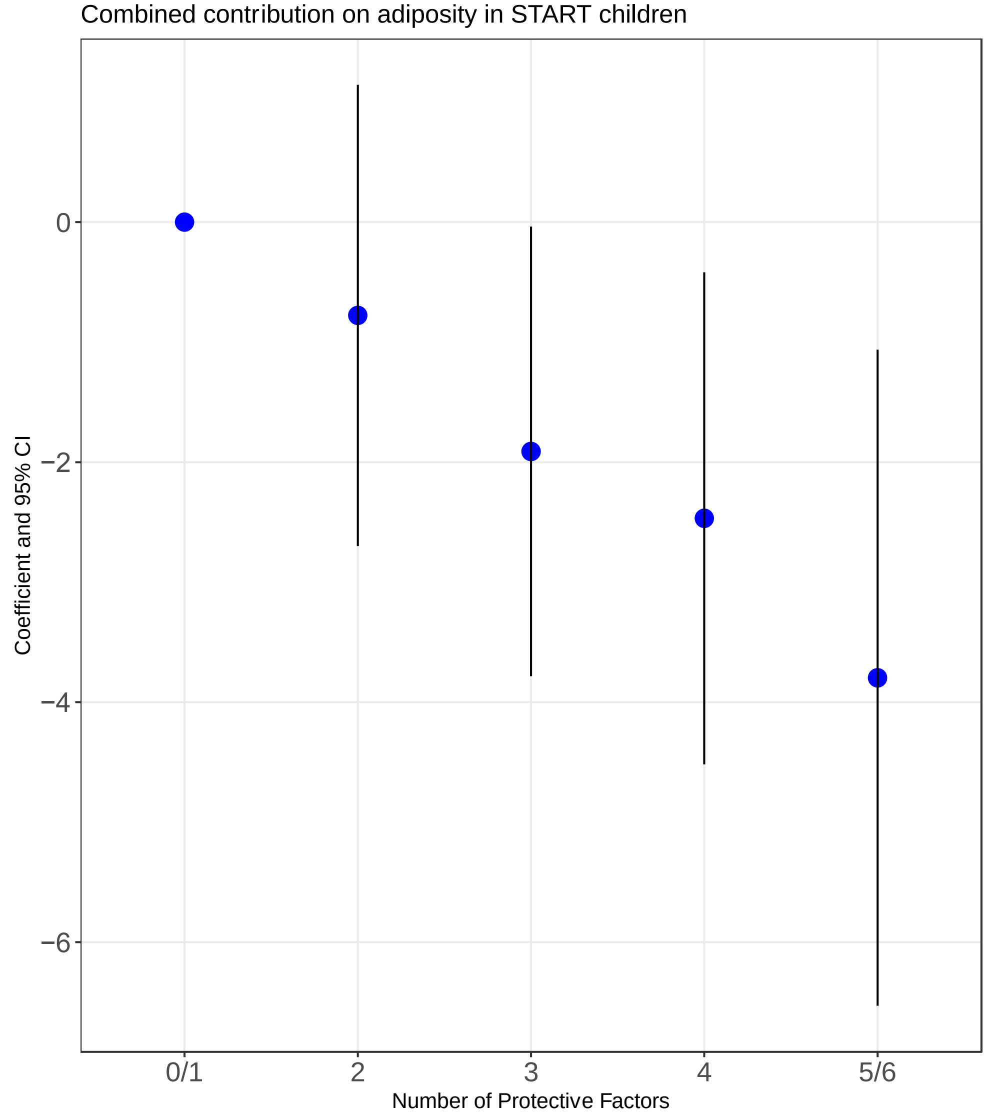


**eFigure 3: Combined contribution on adiposity in children in START, based on clinical recommendation guided cut-offs.** Coefficients and 95% CI for adiposity ($SSF_{AUC}$), adjusting for child sex and age at the 3 yr visit, according to the number/score of modifiable protective factors. Score was calculated as follows: one point for: ≤ 23 of maternal BMI, not excessive gestational weight gain, ≥ median of maternal health-conscious diet score, breastfeeding for 1 yr, ≥ 180 min of physical activity, ≤ 1hr of screen time exposure.

© 2025 Azab SM et al. *JAMA Network Open*.

## eMethods:

## Growth trajectories across the first three years of life:

The primary outcome of our study was the overall trajectory of adiposity i.e. SSF from birth to 3 years of age. In the discovery cohort, the overall trajectory of BMI from birth to 3 years of age was also derived as a comparator to SSF. To capture change over time, we calculated the cumulative area under the curve (AUC) for each child's growth curve of SSF and BMI from birth to 3 years. We used trapezoid calculations to derive the mean of two anthropometric values at two successive visits, multiplied by the time difference between the two visits, for the three time periods (birth to year 1 visit; year 1 to year 2 visit; and year 2 to year 3 visit). The resulting three trapezoid areas were summed to obtain a continuous $SSF_{AUC}$ (mm*year) and $BMI_{AUC}$ ($m^2$/kg*year) value for each participant which reflects the trend over the first three years of life, as outlined in the following formula:

AUC =

Average (SSF or BMI at year 1 visit, SSF or BMI at birth) * (Age at year 1 visit – 0)

+

Average (SSF or BMI at year 2 visit, SSF or BMI at year 1 visit) * (Age at year 2 visit– Age at year 1 visit)

+

Average (SSF or BMI at year 3 visit, SSF or BMI at year 2 visit) * (Age at year 3 visit – Age at year 2 visit)

Similarly, $SSF_{AUC}$ was calculated for BiB as the sum of five trapezoids using birth and five follow-up timepoints (0.5, 1, 1.5, 2, and 3 years). Thus, measurements at 4 to 6 different timepoints were used to derive $SSF_{AUC,}$ and $BMI_{AUC}$. We restricted analysis to children who were 3 years old +/- 6 months at the time of the final 3-year visit, and who had completed at least one follow-up visit (Figure S1). For those children who did not have measurements at all the timepoints, we used an iterative imputation method based on a random forest (missForest R package) to derive missing SSF and BMI values, using pertinent maternal and delivery variables, and child anthropometric and feeding variables, to inform the model.[1] As data quality checks, comparing to children with no missing data, no unexpected outliers were caused by the imputation process and excluding those children with 2 or more missing points had minimal impact on the overall $SSF_{AUC,}$ and $BMI_{AUC}$ distribution (eFigure 2).

The purpose of using AUC was the use of a simplified approach as a crude continuous metric of a child's growth trajectory summarizing serial measurements into a single value.[2] The AUC method is a two-stage method which in the 1st stage identified the AUC as a suitable summary measure of adiposity trajectory calculated for each participant. In the 2nd stage, the summary measure is analyzed by simple statistical techniques as though they were raw data.[3] Mathematically, the area under the curve is calculated by using smaller windows (area = width * height) because the curve shifts up and down uniquely for each individual. Once aggregated into AUC, there is no way of separating it at the different timepoints and the metric becomes a novel outcome to reflect the trajectory of adiposity over a 3-year timespan constructing a latent measure of early life adiposity for each subject.

Most studies have used z-BMI to classify childhood obesity, while other adiposity measures of adiposity such as skinfold thicknesses are less studied.[4] Moreover, there is a wealth of studies focusing on a single timepoint to assess childhood obesity e.g., at birth, at 3 months, at 6 years, etc. but only a few attempted to capture a growth trajectory over time. Adair et al investigated linear growth using conditional weight gain that represented a child's deviation from expected growth based on the child's prior size.[5] Others used latent growth curve analysis or categorized z-BMI trajectories from birth to age four into rising-high-, moderate- and low-BMI.[6,7] Compared to more advanced statistical techniques, the AUC method is easy to understand and easy to use.[3] It also alleviates the need to choose an appropriate correlation structure which can be a complex step associated with other methods such as generalized estimating equations. The $SSF_{AUC}$ metric we adopted in this study as a proxy for the trajectory of adiposity across the early years of life may serve as a useful approach for other researchers and future investigations.

## Maternal exposure variables

We investigated established maternal factors that influence the development of adiposity in the offspring. Maternal exposure variables were defined as: age at pregnancy (years); pre-pregnancy BMI calculated at enrolment (2nd trimester) using measured height and self-reported pre-pregnancy weight (kg/$m^2$); SSF at enrolment as a surrogate measure of maternal adiposity (mm); gestational weight gain calculated by subtracting self-reported pre-pregnancy

© 2025 Azab SM et al. *JAMA Network Open*.

weight from the weight measured immediately before delivery (kg); glucose tolerance in pregnancy from a 75-g oral glucose tolerance test (OGTT) administered at enrolment, estimated as the AUC of the glucose curve (AUC glucose) using fasting plasma glucose, 1 hr post-load glucose, and 2 hr glucose levels [8] (mmol*min); gestational diabetes as previously defined[9]; gestational hypertension; maternal dietary patterns were derived from a validated food frequency questionnaire (FFQ) principal component analysis[10]. The FFQs were specifically designed for South Asians and White Europeans, and captured ethnicity-specific foods, and have been harmonized between START and FAMILY.[11] The three identified dietary patterns were "plant-based," "Western," and "health-conscious"[11]; self-reported smoking history at enrolment categorized as never smoked, quit before pregnancy, quit during pregnancy, and current smoker; socioeconomic status as captured with a validated social disadvantage index composed of household income, marital status and maternal employment (score from 0 to 5)[12]; and physical activity levels obtained from standardized questionnaires including sports and out-of-breath exercise (*e.g.*, walking, jogging) duration (min/d) prior to and during pregnancy. In BiB, pre-pregnancy weight was not recorded, and first trimester maternal BMI was used as a proxy for maternal adiposity, while gestational weight gain and maternal dietary patterns could not be derived.

## Infancy and childhood exposure variables

Based on existing literature and prior knowledge, early life factors affecting childhood adiposity and obesity were selected. Infancy and childhood exposure variables were collected using a combination of measurements at delivery and questionnaires administered to the mothers during follow-up visits, as follows: gestational age; size for gestational age where infants born at 37 weeks' gestation or later were classified by gestational age and sex-specific cut-points as large for gestational age (≥ 90th percentile) or small for gestational age (< 10th percentile); infant biological sex; breastfeeding status at 1-year defined as whether or not child was breastfed up to the first year of life irrespective of breastfeeding exclusivity; age of solid food introduction (months); parent-reported night time sleep calculated as a cumulative average of year 1, 2, and 3; screen time exposure (television, computer, tablet, mobile device, video games) calculated as a cumulative average of year 2 and 3 (hr/day); and ultra-processed food consumption using the NOVA classification system, and derived from dietary information collected using a semiquantitative FFQ completed by the mother for her child and calculated as a cumulative average of year 1 and 2 (% of total daily servings). Child's physical activity calculated as a cumulative average of time child spent engaged in physically challenging activities (*e.g.*, playground, running, on the bike inside or outside the house) in year 1, 2, and 3 in START (min/day); in FAMILY, the mother estimated the percentage of time in a day her child spent as: (a) inactive: sleeping, lying down, resting, napping; (b) somewhat inactive: sitting, reading, watching TV, playing video games, playing quiet games, or activities which are mostly done sitting down; (c) somewhat active: walking, climbing stairs, household chores; and (d) active: running, jumping, skipping, bicycling, skating, swimming, skipping, and a weighted sum of the somewhat active and active categories was derived and thereof a cumulative average of this physical activity variable in year 1, 2, and 3 (min/day)[13]; in BiB, the mother estimated how long and how often per week her child spent playing actively inside the house and in the garden and a cumulative average of this physical activity variable in year 2 and 3 (min/day) was derived.

## Clinically guided cut-offs for deriving the combined score

A secondary analysis was conducted in the discovery cohort START, using clinical recommendation guided cut-offs, instead of the median, to derive the combined score as follows:

- one point for: maternal BMI ≤ 23 (South Asian cutoff for overweight)
- one point for: not excessive gestational weight gain (The Institute of Medicine (IOM) recommendations)
- one point for: ≥ median of maternal health-conscious diet score
- one point for: breastfeeding for 1 yr
- one point for: ≥ 180min of physical activity (Canadian Society for Exercise Participaction guidelines)
- one point for: ≤ 1 hour of screen time exposure (American Pediatrics Association guidelines).

## eReferences:


1. Stekhoven DJ, Buhlmann P. MissForest--non-parametric missing value imputation for mixed-type data. *Bioinformatics*. 2012;28(1):112-118. doi:10.1093/bioinformatics/btr597

2. Rafiq T, Stearns JC, Shanmuganathan M, et al. Integrative multiomics analysis of infant gut microbiome and serum metabolome reveals key molecular biomarkers of early onset childhood obesity. *Heliyon*. 2023;9(6):e16651. doi:10.1016/j.heliyon.2023.e16651

3. Matthews JN, Altman DG, Campbell MJ, Royston P. Analysis of serial measurements in medical research. *BMJ*. 1990;300(6719):230-235. doi:10.1136/bmj.300.6719.230

4. Azab SM, Shanmuganathan M, De Souza RJ, et al. Early sex-dependent differences in metabolic profiles of overweight and adiposity in young children: a cross-sectional analysis. *BMC Med*. 2023;21(1):176. doi:10.1186/s12916-023-02886-8

5. Adair LS, Fall CH, Osmond C, et al. Associations of linear growth and relative weight gain during early life with adult health and human capital in countries of low and middle income: findings from five birth cohort studies. *The Lancet*. 2013;382(9891):525-534. doi:10.1016/S0140-6736(13)60103-8

6. Coppens E, Bardid F, Deconinck FJA, et al. Developmental Change in Motor Competence: A Latent Growth Curve Analysis. *Front Physiol*. 2019;10:1273. doi:10.3389/fphys.2019.01273

7. Zhao Q, Hu Z, Kocak M, et al. Associations of prenatal metabolomics profiles with early childhood growth trajectories and obesity risk in African Americans: the CANDLE study. *Int J Obes*. 2021;45(7):1439-1447. doi:10.1038/s41366-021-00808-3

8. Anand SS, Gupta M, Teo KK, et al. Causes and consequences of gestational diabetes in South Asians living in Canada: results from a prospective cohort study. *CMAJ Open*. 2017;5(3):E604-E611. doi:10.9778/cmajo.20170027

9. Sikorski C, Azab S, de Souza RJ, et al. Serum metabolomic signatures of gestational diabetes in South Asian and white European women. *BMJ Open Diabetes Res Care*. 2022;10(2):e002733. doi:10.1136/bmjdrc-2021-002733

10. Kelemen LE, Anand SS, Vuksan V, et al. Development and evaluation of cultural food frequency questionnaires for South Asians, Chinese, and Europeans in North America. *J Am Diet Assoc*. 2003;103(9):1178-1184. doi:10.1016/S0002-8223(03)00985-4

11. de Souza RJ, Zulyniak MA, et al. Harmonization of Food-Frequency Questionnaires and Dietary Pattern Analysis in 4 Ethnically Diverse Birth Cohorts. *J Nutr*. 2016;146(11):2343-2350. doi:10.3945/jn.116.236729

12. Anand SS, Razak F, Davis A, et al. Social disadvantage and cardiovascular disease: development of an index and analysis of age, sex, and ethnicity effects. *Int J Epidemiol*. 2006;35(5):1239-1245. doi:10.1093/ije/dyl163

13. Azab SM, de Souza RJ, Lamri A, et al. Metabolite profiles and the risk of metabolic syndrome in early childhood: a case-control study. *BMC Med*. 2021;19(1):292. doi:10.1186/s12916-021-02162-7

© 2025 Azab SM et al. *JAMA Network Open*.
