# Supplementary material for: Trajectory of Early Life Adiposity Among South Asian Children
Source: JAMA Netw Open. 2025 Apr 10;8(4):e254439. doi: 10.1001/jamanetworkopen.2025.4439 (PMC11986779; doi:10.1001/jamanetworkopen.2025.4439)
Supplement: Supplement 2. — Data Sharing Statement [file jamanetwopen-e254439-s002.pdf]

Data Sharing Statement

Azab. Trajectory of Early Life Adiposity Among South Asian Children. *JAMA Netw Open*. Published April 10, 2025. doi:10.1001/jamanetworkopen.2025.4439

Data
Data available: No

Additional Information
Explanation for why data not available: The participants in the studies used for this paper did not consent to making the data publicly available. However, the data can be made available upon reasonable request to the corresponding author.
